# A randomized, controlled trial on the effects of almonds on lipoprotein response to a higher carbohydrate, lower fat diet in men and women with abdominal adiposity

**DOI:** 10.1186/s12944-019-1025-4

**Published:** 2019-04-03

**Authors:** Paul T. Williams, Nathalie Bergeron, Sally Chiu, Ronald M. Krauss

**Affiliations:** 1 Children’s Hospital Oakland Research Institute, Oakland, California USA; 2 College of Pharmacy, Touro University California, Vallejo, California USA

**Keywords:** Almonds, Triglycerides, Lipids, Lipoprotein size, High carbohydrate

## Abstract

**Background:**

Almonds have been shown to lower LDL cholesterol but there is limited information regarding their effects on the dyslipidemia characterized by increased levels of very low density lipoproteins (VLDL) and small, dense low-density lipoprotein (LDL) particles that is associated with abdominal adiposity and high carbohydrate intake. The objective of the present study was to test whether substitution of almonds for other foods attenuates carbohydrate-induced increases in small, dense LDL in individuals with increased abdominal adiposity.

**Methods:**

This was a randomized cross-over study of three 3wk diets, separated by 2wk washouts: a higher-carbohydrate (CHO) reference diet (CHO_high_), a higher-CHO diet with isocaloric substitution of 20% kcal (E) from almonds (CHO_high + almonds_), and a lower-CHO reference diet (CHO_low_) in 9 men and 15 women who were overweight or obese. The two CHO_high_ diets contained 50% carbohydrate, 15% protein, 35% fat (6% saturated, 21% monounsaturated, 8% polyunsaturated), while the CHO_low_ diet contained 25% carbohydrate, 28% protein, 47% fat (8% saturated, 28% monounsaturated, 8% polyunsaturated). Lipoprotein subfraction concentrations were measured by ion mobility.

**Results:**

Relative to the CHO_low_ diet: 1) the CHO_high + almonds_ diet significantly increased small, dense LDLIIIa (mean difference ± SE: 28.6 ± 10.4 nmol/L, *P* = 0.008), and reduced LDL-peak diameter (− 1.7 ± 0.6 Å, P = 0.008); 2) the CHO_high_ diet significantly increased medium-sized LDLIIb (24.8 ± 11.4 nmol/L, *P* = 0.04) and large VLDL (3.7 ± 1.8 nmol/L, *P* = 0.05). Relative to CHO_low_, the effects of CHO_high_ on LDLIIIa (17.7 ± 10.6 nmol/L) and LDL-peak diameter (− 1.1 ± 0.6 Å) were consistent with those of CHO_high + almonds_, and the effects of CHO_high + almonds_ on LDLIIb (21.0 ± 11.2 nmol/L) and large VLDL (2.8 ± 1.8 nmol/L) were consistent with those of CHO_high_, but did not achieve statistical significance (*P* > 0.05). None of the variables examined showed a significant difference between the CHO_high + almonds_ and CHO_high_ diets (*P* > 0.05).

**Conclusion:**

Our analyses provided no evidence that deriving 20% E from almonds significantly modifies increases in levels of small, dense LDL or other plasma lipoprotein changes induced by a higher carbohydrate low saturated fat diet in individuals with increased abdominal adiposity.

**Trial registration:**

Clinicaltrials.gov NCT01792648.

**Electronic supplementary material:**

The online version of this article (10.1186/s12944-019-1025-4) contains supplementary material, which is available to authorized users.

## Background

Almonds appear to promote healthy blood lipid and lipoprotein levels [1]. A recent meta-analysis of randomized controlled clinical trials found that almond consumption reduced plasma total cholesterol concentrations by 0.15 mmol/L , LDL-cholesterol concentrations by 0.12 mmol/L, and triglyceride concentrations by 0.07 mmol/L [2]. Moreover, a meta-analysis of prospective epidemiologic studies reported that increased consumption of almonds and other nuts was associated with a substantial reduction in CVD mortality (RR 0.73; 95% CI 0.68–0.78) for the highest vs lowest quintile of intake [3].

Low density lipoproteins include multiple subclasses that range from small, dense, lipid-depleted LDL particles to large, buoyant, cholesterol-enriched LDL particles [4]. Small LDL particles have been shown to be more strongly associated with increased CVD risk than larger LDL [5–7]. High plasma concentrations of small LDL particles and triglyceride, and low HDL-cholesterol concentrations define atherogenic dyslipidemia [8]. This dyslipidemia is one component of metabolic syndrome, which also includes abdominal obesity (defined by increased waist circumference), and/or dysglycemia, and/or high blood pressure.

The optimal dietary macronutrient distribution for improving blood lipids and overall CVD risk may differ across individuals. Replacement of dietary total and saturated fat with carbohydrates effectively lowers total and LDL-cholesterol [9]. However, we have shown that in most healthy individuals, low fat, high carbohydrate diets do not result in overall improvements in lipoprotein profiles and can instead increase plasma triglycerides and small, dense LDL particle concentration [10–12]. Analysis of combined data from several dietary intervention studies, in which carbohydrate and fat intakes varied inversely over a broad range and where protein intake was constant, revealed a strong linear relationship between increasing carbohydrate intake and the atherogenic lipoprotein phenotype defined by high concentrations of small dense LDL particles [9].

A triglyceride-lowering effect of almonds may be especially beneficial to the approximately one-third of Americans with metabolic syndrome [13], particularly if levels of small LDL are also reduced with almond supplementation (triglycerides and small LDL being concordantly related [8]). While increasing evidence supports the cardiometabolic benefit of restricting dietary carbohydrates in individuals with the atherogenic dyslipidemia of metabolic syndrome, for those unwilling to do so, we sought to test whether almond consumption can reduce levels of small and medium LDL particles without the need to restrict dietary carbohydrates to levels below those currently recommended.

## Methods

### Participants and recruitment

The study was conducted between July 2012 and January 2016 at the Cholesterol Research Center (Berkeley, CA). Twenty four men and women were recruited from participants in our previous dietary studies and from the local community through internet and newspaper advertisements, outreach programs, bulk mailing and referrals through collaborations with other academic or corporate institutions. Participants who passed a self-administered web-based pre-screening questionnaire were contacted by a recruiter to review the eligibility requirements and study protocol, and by the study nutritionist to discuss the dietary requirements. Those who agreed to the study protocol received a clinical assessment and blood draw during their initial screening visit. This study was conducted according to the guidelines laid down in the Declaration of Helsinki and the procedures involving human subjects were approved by the Children’s Hospital and Research Center Oakland Institutional Review Board. Written informed consent was obtained from all subjects. This clinical trial was registered on ClinicalTrials.gov under the identifier NCT01792648 (https://clinicaltrials.gov/ct2/show/NCT01792648).

Our initial intent was to study individuals with atherogenic dyslipidemia of the metabolic syndrome, defined by triglycerides ≥1.69 mmol/L, HDL-cholesterol  < 1.03 mmol/L (men) or < 1.29 mmol/L (women), and with at least one additional characteristic of metabolic syndrome (waist circumference > 102 cm (men) or > 88 cm (women), blood pressure ≥ 130/≥85 mmHg, or fasting glucose ≥6.1 mmol/L) [14]. Of the screened participants who had triglycerides > 1.69 mmol/L, only ~ 65% had low HDL-cholesterol levels consistent with atherogenic dyslipidemia. Of these, less than 10% had one additional criterion for metabolic syndrome and were willing to take part in the study. Such low prevalence of atherogenic dyslipidemia with features of the metabolic syndrome may be a consequence of our stringent enrollment criteria which excluded individuals taking lipid- or glucose-lowering medications, smokers, and the presence of comorbidities. As such, and after sustained difficulty identifying participants who met these criteria, only abdominal obesity (waist circumference > 88 cm for women and > 102 cm for men) was retained as the selection criterion. Of the 24 participants who completed the study, 2 did not meet the waist criterion but had other characteristics of metabolic syndrome. Exclusion of these two participants from data analysis did not affect any of the parameters measured (lipids, lipoproteins, blood pressure, insulin, glucose, inflammatory markers). Changes to the enrollment criteria were approved by our Institutional Review Board.

Additional screening inclusion criteria included: 1) ≥ 20 years; 2) no history of coronary heart disease, cerebrovascular disease, peripheral vascular disease, bleeding disorder, liver or renal disease, diabetes, lung disease, HIV, or cancer (other than skin cancer) in the last 5 years; 3) not pregnant or breast feeding, and agreeing to use appropriate barrier contraception throughout the study for women of childbearing potential; 4) no current use of hormones or drugs known to affect lipid metabolism or blood pressure; 5) no current use of nicotine products or recreational drugs; 6) willingness to abstain from alcohol or dietary supplements during the study; 7) systolic blood pressure < 160 mmHg and diastolic blood pressure < 95 mmHg; 8) body mass index (BMI) ≤ 38 kg/m^2^; 9) total- and LDL-cholesterol  < 95th percentile for sex and age, 10) fasting triglycerides > 0.56 mmol/L and < 5.65 mmol/L, 11) fasting blood sugar < 7.0 mmol/L ; 12) thyroid stimulating hormone (TSH) within normal range; and 13) weight stable for > 3 months.

### Experimental design and setting of the study

The diet intervention was carried out on an outpatient basis with careful monitoring of compliance. In a randomized crossover design, each participant consumed the three experimental diets (a higher-carbohydrate (CHO) reference diet (CHO_high_); a higher-CHO diet with almonds (CHO_high + almonds_); and a lower-CHO reference diet (CHO_low_)) for 3 weeks each, separated by 2-week washout periods during which participants consumed their habitual diet (see Additional file 1: Table S1). Diet assignments were kept in sequentially numbered sealed envelopes and assigned to the participant by the clinic staff 1–2 days before starting the intervention. Investigators and laboratory staff were blinded to diet assignment, while clinic staff were not. Participants were not informed of their diet assignment, but due to the nature of the experimental diets, they were likely able to identify them. The three experimental diets consisted of a higher carbohydrate reference diet (CHO_high_ diet: 50% E carbohydrate; 15% protein; 35% total fat); an almond-supplemented (20% E) diet with similar macronutrient composition to the higher carbohydrate reference diet (CHO_high + almond_ diet: 50% E carbohydrate; 15% protein; 35% total fat); and a lower carbohydrate reference diet (CHO_low_: 25% E carbohydrate; 28% protein; 47% total fat). The CHO_high_ diet followed current dietary recommendations that focus on restricting saturated fat and increasing intake of unsaturated fat (Table 1). The CHO_high + almond_ diet provided 20% E from almonds in partial replacement for cooking oils and other fat sources (avocados, olives, etc.), with all other foods being otherwise comparable among the standard reference and almond-supplemented diets. Almonds (nonpareil variety) were provided by the Almond Board of California. They were distributed to participants in pre-weighed packets when consumed as raw whole unsalted almonds (10% E), with the remaining almonds (10% E) consumed as raw almond meal integrated in customized unit foods (a choice of either roasted pepper almond spread, almond soup or almond cake). For both the CHO_high_ and CHO_high + almond_ diets, the ratio of starch to total sugars was 60/40, with fruit and dairy products constituting the bulk of total sugars, and added sugars representing 6% of daily calories. The composition of the CHO_low_ diet was based on our earlier findings that more extreme carbohydrate restriction, from 54% E to 26% E, is required to significantly reduce LDL-cholesterol as well as small and very small LDL particles, whereas more moderate carbohydrate restriction (39% E) failed to significantly reduce LDL-cholesterol or its component subclasses [15]. For the CHO_low_ diet, the ratio of starch to total sugars was 70/30, with fruit constituting the bulk of total sugars and added sugars representing < 3% of daily calories. During the washout period the participants consumed their usual home diets. Comparable amounts of total fat in the standard reference and almond-supplemented diets were achieved primarily by limiting intake of foods rich in unsaturated fat and partially replacing cooking oils with almonds.

**Table 1:** Composition of experimental diets^a^

	CHO_high + almond_	CHO_high_	CHO_low_
Carbohydrate, % kcal	50	50	25
Protein, % kcal	15	15	28
Total fat, % kcal	35	35	47
SFA	6	6	8
MUFA	21	21	28
PUFA	8	8	8
Cholesterol (mg/d)	218	257	265
Fiber (g/d)	39	32	31

^a^Calculated values (Nutrition Data System for Research software 2010) for 2500 kcal 3-day menus. *MUFA* monounsaturated fatty acids, *PUFA* polyunsaturated fatty acids

Participants were provided ~ 65% daily energy in the form of two frozen entrees (lunch and dinner) and snacks. In addition, participants received detailed dietary instructions, standardized menus, checklists, and shopping lists for home preparation of breakfast and sides for the remaining food items on the menu. Itemized grocery receipts were collected regularly from participants to verify their purchase of perishable foods on their shopping list. Participants were instructed to eat all food items provided/prescribed, and to report any deviations from the protocol. A compliance score (1–5-point scale, where 5 is indicative of high compliance) was assigned to each study participant by the staff nutritionist, based on menu checklists, itemized grocery receipts and information gathered from weekly interactions.

The nutrient composition of the diets was assessed using ProNutra software (Viocare Technologies, Inc.) and the Nutrition Data System for Research (University of Minnesota). Three-day rotating menus were provided at four calorie levels (2000, 2500, 3000, 3500 kcal) for each of the diets, and snacks (200–350 kcal) similar in nutrient composition to the experimental diets were provided for individuals whose calorie needs were intermediate to the 4 calorie levels available. The participants’ energy requirements for maintaining stable weight were estimated using the Institute of Medicine equation [16]. During the study, participants were required to maintain their body weight within ±3% of their initial weight over the course of any consecutive two weeks.

Height and weight were measured during the clinic visits. Participants wore a pedometer to monitor daily steps. Baseline steps per day were measured during the study run-in period and participants were asked to maintain this level of activity throughout the study. Number of steps and other physical activities were recorded in logs and reviewed during weekly diet visits. Fasting blood samples were collected at the end of each dietary period for measurement of plasma lipids, lipoproteins, lipoprotein subfractions, and apolipoproteins B and AI, as well as markers of insulin resistance and inflammation after fasting overnight for 12–14 h. Plasma was separated immediately by centrifugation at 4 °C. Including the screening visit, participants visited the clinic for a total of 7 blood draws and met with the research nutritionist weekly on 14 separate occasions.

### Laboratory measurements

Plasma triglycerides, total- and HDL-cholesterol were measured by enzymatic endpoint analysis on a clinical chemistry analyzer (Liasys 330) using methodology previously described [17–19]. Triglyceride and cholesterol measurements are standardized through the CDC-NHLBI lipid standardization program. LDL-cholesterol was calculated from the Friedewald equation [20]. ApoB and apoAI were determined by immunoturbidimetric assay using the ITA reagent kit [21, 22]. Plasma particle concentrations of VLDL, intermediate density lipoprotein (IDL), LDL and HDL subfractions were analyzed by gas-phase electrophoresis (i.e., ion mobility [23, 24]). Inter-assay variations of the subfraction measurements were minimized by the inclusion of two in-house controls in each preparatory process and by duplicate analysis (CV < 15%).

Fasting glucose was measured by enzymatic endpoint analysis and fasting insulin by ELISA (EZHI-14 K Human Insulin ELISA kit; Millipore, Billeria, MA, USA), with two in-house quality control standards. HOMA-IR was calculated as a marker of insulin resistance (insulin (μU/mL) x glucose (mg/dL)/405) [25]. Plasma samples were assayed for a multi-analyte panel of inflammatory biomarkers (interleukins 1, 6, 8, and 10, resistin, TNF-alpha, PAI-1, leptin, MCP, SAA, lipocalin, and BAFF) using a multiplex immunoassay method through a CLIA certified commercial laboratory (Rules Based Medicine Austin, TX).

### Statistical analyses

Randomization of the dietary sequence within random size blocks was performed by the statistician assigned to the study using a uniform random-number generator. Treatment, sequence, period and carryover effects were calculated using ANOVA for a cross-over design with the statistical package Stata (version 11, Austin, TX). The primary hypotheses were tested by comparing the effects of the almond-supplemented diet (20% E) with those of the two reference diets that did not contain almond products: one with similar content of carbohydrate, protein, and total, saturated, monounsaturated, and polyunsaturated fat (CHO_high_), and the other in which carbohydrate content was substantially reduced by substitution of protein and monounsaturated fat (CHO_low_). The CHO_high_ diet was compared to the CHO_low_ diet to ensure that the study design, interventions and duration of the experimental diets and washout periods produced the expected diet-induced changes. The primary hypothesis was that almond supplementation would significantly attenuate the increase in small, dense LDL and apolipoprotein B relative to the CHO_high_ standard reference diet while maintaining or enhancing the reduction in total cholesterol and LDL-cholesterol relative to a CHO_low_ diet. Secondary hypotheses included the effects of these dietary interventions on total: HDL-cholesterol ratio, LDL peak particle size, triglycerides, HDL measures (HDL-cholesterol, HDL subclasses, apoAI), and insulin resistance as estimated by HOMA-IR, as well as a panel of inflammatory biomarkers. The study was initially designed to detect a 5.3 mg/dL difference in LDL-cholesterol reduction between the CHO_hig__h__ + almond_ diet and CHO_high_ standard diet in 40 subjects. This detectable difference is smaller than the expected 9.5 mg/dl reduction in LDL-cholesterol for 20% E from almonds calculated in the meta-analysis by Sabate et al. [26]. Interim analyses determined midway through the study that almond supplementation would be highly unlikely to improve LDL-cholesterol levels by the study’s completion. Recruitment was therefore terminated at 24 subjects. Specifically, even if the remaining 16 subjects all had LDL-cholesterol differences of 9.5 mg/dL, we still would not have achieved the 5.3 mg/dL difference between diets which the study was designed to detect.

## Results

**Fig. 1:**
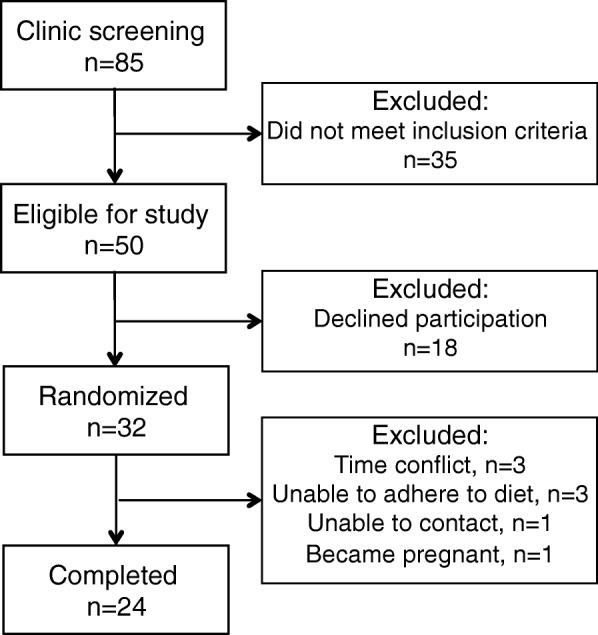
Participant enrollment

Figure 1 shows the details of participant recruitment and enrollment. Table 2 presents the baseline characteristics of the study participants. Fifteen participants (62.5%) had total cholesterol ≥5.17 mmol/L, 17 (71%) had LDL-cholesterol ≥2.59 mmol/L, 9 patients (37.5%) had triglycerides ≥1.69 mmol/L, 7 patients (29.1%) had HDL-cholesterol < 1.03 mmol/L if male and < 1.29 mmol/L if female, and all but 1 patient had at least one screening lipid value outside the desirable range according to the National Cholesterol Education Program-III guidelines. One-third of the participants had fasting plasma glucose > 6.1 mmol/L.

**Table 2:** Screening characteristics of the study participants

	Males (*N* = 9)		Females (*N* = 15)	
	Mean	SD	Mean	SD
Age, y	41	15	52	9
Body mass index (kg/m^2^)	31.7	4.2	31.0	3.3
Waist circumference (cm)	106.8	10.0	99.4	7.9
Systolic blood pressure (mmHg)	130	13	122	17
Diastolic blood pressure (mmHg)	75	8	71	10
Triglycerides (mmol/L)	1.76	0.34	1.39	0.68
Total cholesterol (mmol/L)	4.61	0.50	5.29	0.61
LDL-cholesterol (mmol/L)	2.79	0.43	2.92	0.60
HDL-cholesterol (mmol/L)	1.01	0.21	1.73	0.47
Total−/HDL-cholesterol	4.6	0.6	3.2	0.8
Glucose (mmol/L)	5.9	0.4	5.9	0.5

Participants were compliant with the dietary protocol, with nutritionist-rated scores, on a scale of 1 to 5, averaging 4.5 ± 0.9 (SD); 4.6 ± 0.8 and 4.4 ± 0.8, for the CHO_high_, CHO_high + almond_ and CHO_low_ diets, respectively.

The overall significance levels for the effects of treatment, period, sequence, and carryforward are presented in Additional file 1: Table S2. Overall treatment effects were significant for diastolic blood pressure (*P* = 0.01) and medium-sized LDLIIb (*P* = 0.02). Period and carryforward effects were significant for diastolic blood pressure (*p* = 0.02 for both), large LDLIIa (*P* = 0.03 and *P* = 0.02, respectively), and large LDLI (*P* = 0.05 and *P* = 0.02, respectively). There were no significant sequence effects.

Table 3 presents the differences in the primary outcome variables after completing each diet (the mean levels after each diet are presented in Additional file 1: Table S3). There were no significant weight changes on any of the diets. There were no significant differences in LDL-cholesterol or small to medium LDL subclasses between the CHO_high + almonds_ diet and the CHO_high_ diet (the primary hypothesis). With 95% confidence, the difference between the CHO_high + almonds_ vs. CHO_high_ diet was between − 0.18 and 0.23 mmol/L for LDL-cholesterol and between − 11.08 and 33.07 nmol/L for LDL IIIa.

**Table 3:** Mean differences in weight, blood pressure, lipoproteins and insulin resistance between diets

	Mean and SE for differences between diets					
	CHO_High + almonds_-CHO_High_		CHO_High + almonds_- CHO_Low_		CHO_High_-CHO_Low_	
	Mean	SE	Mean	SE	Mean	SE
Body mass (kg)	−0.1	0.4	0.4	0.3	0.6	0.4
Body mass index (kg/m^2^)	− 0.1	0.1	0.1	0.1	0.2	0.1
Waist circumference (cm)	0.4	0.6	0.7	0.6	0.3	0.6
Systolic BP (mmHg)	−1	1	1	1	2	1
Diastolic BP (mmHg)	−1	1	0	1	1	1
Triglycerides (mmol/L)	0.03	0.20	0.02	0.20	−0.02	0.20
Total cholesterol (mmol/L)	−0.05	0.10	0.11	0.10	0.16	0.10
LDL-cholesterol (mmol/L)	0.02	0.10	0.04	0.10	0.02	0.10
HDL-cholesterol (mmol/L	−0.08	0.06	−0.01	0.06	0.07	0.06
nonHDL-cholesterol (mmol/L)	0.02	0.12	0.11	0.11	0.09	0.11
Total−/HDL-cholesterol	0.0	0.1	0.1	0.1	0.0	0.1
Glucose (mmol/L)	0.1	0.1	−0.1	0.1	−0.1	0.1
Insulin (pmol/L)	1.6	1.7	0.6	1.7	−1.1	1.7
HOMA-IR	0.4	0.5	0.13	0.46	−0.30	0.46
Apolipoprotein AI (g/L)	0.01	0.02	0.03	0.02	0.03	0.02
Apolipoprotein B (g/L)	−0.05	0.04	−0.01	0.04	0.04	0.04
HDL3&2a (nmol/L)	100	400	569	397	469	405
HDL2b (nmol/L)	− 267	327	−18	316	249	323
LDL IVc (nmol/L)	−0.6	2.6	2.5	2.6	2.5	2.6
LDL IVb (nmol/L)	3.9	5.2	7.7	5.0	3.8	5.1
LDL IVa (nmol/L)	8.7	10.6	14.0	10.3	5.3	10.3
LDL IIIb (nmol/L)	7.5	5.8	10.3	5.6	2.8	5.76
LDL IIIa (nmol/L)	11.0	10.7	28.6†	10.4	17.7	10.6
LDL IIb (nmol/L)	−3.8	11.5	21.0	11.2	24.8*	11.4
LDL IIa (nmol/L)	−7.0	11.2	0.2	10.8	7.2	11.1
LDL I (nmol/L)	−1.8	16.4	−19.7	16.2	−19.7	15.2
IDL2 (nmol/L)	5.9	10.5	−5.8	10.1	−11.7	10.4
IDL1 (nmol/L)	4.2	7.3	11.3	7.0	7.0	7.2
Small VLDL (nmol/L)	1.4	3.3	3.3	3.2	1.9	3.2
Intermediate VLDL (nmol/L)	−0.7	3.9	6.3	3.8	7.0	3.9
Large VLDL (nmol/L)	−0.9	1.9	3.7	1.8	3.7*	1.8
LDL peak diameter (Å)	−0.6	0.6	−1.7†	0.6	−1.1	0.6

* *P* ≤ 0.05; † *P* ≤ 0.01

With respect to secondary hypotheses, relative to the CHO_low_ diet: 1) the CHO_high + almonds_ diet significantly increased plasma concentrations of small, dense LDLIIIa (mean difference ± SE: 28.6 ± 10.4 nmol/L, *P* = 0.008), and reduced LDL-peak diameter (− 1.7 ± 0.6 Å, *P* = 0.008); and 2) the CHO_high_ diet significantly increased plasma concentrations of medium sized LDLIIb (24.8 ± 11.4 nmol/L, *P* = 0.04) and large VLDL (3.7 ± 1.8 nmol/L, *P* = 0.05). While not achieving statistical significance, the effects of CHO_high_ on LDLIIIa (17.7 ± 10.6 nmol/L, *P* = 0.10) and LDL-peak diameter (− 1.1 ± 0.6 Å, *P* = 0.08) were consistent with those of CHO_high + almonds_, and the effects of CHO_high + almond_ on LDLIIb (21.0 ± 11.2 nmol/L, *P* = 0.07) and large VLDL (2.8 ± 1.8 nmol/L, *P* = 0.13) were consistent with those of CHO_high_, when compared to CHO_low_. None of the lipoprotein variables showed significant differences between the CHO_high + almonds_ and CHO_high_ diets (*p* > 0.05). The results in Table 3 also show no significant differences among the diets for standard plasma lipid measurements, glucose, insulin, HOMA-IR, apolipoproteins A1 and B, and HDL particle concentrations.

The corresponding analyses for differences in interleukin, resistin, tumor necrosis factor alpha (TNF-α), plasminogen activator inhibitor-1 (PAI-1), leptin, monocyte chemotactic protein (MCP), serum amyloid A (SAA), lipocalin, B lymphocyte activating factor (BAFF), and hepatocyte growth factor (HGF) between diets are presented in Additional file 1: Tables S4-S6. Among these inflammatory measures, only a borderline significant increase in interleukin-1 was observed for the CHO_high + almonds_ diet compared to the CHO_low_ diet (4.6 ± 2.3, *P* = 0.05).

## Discussion

Numerous studies have shown significant lowering of plasma and LDL-cholesterol levels when almonds are substituted for carbohydrates in the diet, but little is known of their metabolic effects in the context of higher carbohydrate intake. In the present study we tested whether lipoprotein measures of CVD risk are reduced by almond supplementation in the context of a diet higher in carbohydrate, and with fat and protein content that conform to current guidelines. Our analyses provided no evidence that modifying the standard reference diet (50% E carbohydrates, 15% protein, 35% total fat) with 20% E derived from almonds significantly lowers plasma LDL-cholesterol or total cholesterol. Moreover, increases in small and midsize LDL concentrations and reductions in LDL-peak diameter were essentially the same for the higher carbohydrate reference diet with or without the inclusion of almond-derived products. Both renditions of the higher carbohydrate diet increased LDLIIIa and LDLIIb plasma concentrations and reduced LDL particle diameter relative to the lower-carbohydrate higher-fat diet.

The present findings differ from that of a recent study in 31 overweight and obese individuals in which diets providing almonds (42.5 g/d) or almonds and dark chocolate (42.5 g/d almonds, plus 43 g/d dark chocolate and 18 g/d cocoa powder) reduced LDL-cholesterol, and lowered large LDL (almond diet) and small LDL (almond plus dark chocolate diet) in comparison to an average American diet [27]. Discrepancies with the present findings may reflect differences in the comparator diet which was low in saturated fat (8% E) and high in fiber (31 g/d) in the present study, but high in saturated fat (13% E) and low in fiber (23 g/d) in the study by Lee et al. [27]. These findings suggest that the high unsaturated fatty acid profile of almonds may contribute, at least in part, to their lipid-lowering effects.

Almonds have been found to promote healthy blood lipid levels, manifest as reductions in total cholesterol, LDL-cholesterol and triglycerides [2]. To date, their effects on plasma lipoprotein concentrations have been investigated in eighteen publications that encompass 27 almond vs. control group comparisons, 17 from parallel trials and 10 from cross-over designs [2]. These included studies in healthy subjects, subjects with high cholesterol, and in diabetic patients. Reductions in total- and LDL-cholesterol are reported to be greatest for almond intake ≥45 g and in studies of individuals with non-optimal baseline lipid levels as defined by the National Cholesterol Education Program Adult Treatment Panel III guidelines (total cholesterol ≥200 mg/dl; LDL-cholesterol≥100 mg/dl) [28]. In the current study, all but one patient had at least one screening lipid value outside the desirable range according to the National Cholesterol Education Program-III guidelines. Nevertheless we cannot rule out an effect of almond consumption in patients with metabolic syndrome who display a greater degree of atherogenic dyslipidemia.

Several of the studies that contributed to the above meta-analyses of almonds and lipoproteins [2] included significant weight loss, although they were not among the studies showing the most significant decreases in total cholesterol, LDL-cholesterol, or plasma triglyceride concentrations. In the present study, the subjects were closely monitored to maintain stable weight, which could have diminished improvements in LDL and other lipoproteins.

In accordance with the initial protocol, the study was terminated early when interim analyses showed no indication that almond mitigated the increases in small LDL and triglycerides on a high carbohydrate diet. The 3-week interventions were also shorter than other almond feeding studies (≥4 weeks) [2]. Nevertheless, a 3-week intervention in 24 subjects was sufficient to produce the expected increases in medium- and small-sized LDL particle concentrations associated with increased carbohydrate intake (Table 3). Significant carry forward effects for diastolic blood pressure, LDL-cholesterol, total cholesterol/HDL-cholesterol, apo B, and larger LDL (Supplemental Table 2) suggest that the design could have benefitted from a longer than 2-week washout period, although prior crossover studies had washouts of 2 weeks or less.

Limitations of the study include the small sample size, relatively short feeding duration, and significant carryforward effects which may have contributed to the nonsignificant almond effect on the primary endpoints. In the 24 subjects who completed the study, we showed that the LDL-cholesterol difference between the CHO_high + almonds_ diet and CHO_high_ diet without almonds was 0.02 ± 0.20 mmol/L (mean difference ± 95% confidence interval) which excludes the 0.25 mmol/L average reduction in LDL-cholesterol for 20% E from almonds reported by Sabate et al. [26]. The LDL-cholesterol difference was nonsignificant when adjusted for carryforward effect (*P* = 0. 38) and the carryforward effect did not prevent the expected increase in LDLIIb for the high vs. low fat diet. Addition of the 24 participants studied here to the 837 included in a meta-analysis of almond effects on plasma lipoproteins [2] would be unlikely to alter the conclusion that “consumption of nuts as part of a healthy diet should be encouraged to help in the maintenance of healthy blood lipid levels and to reduce the risk of heart disease”.

## Conclusion

The absence of differences in plasma LDL-cholesterol and small dense LDL (primary endpoints), and other lipoproteins (secondary endpoints) with CHO_high + almonds_ vs. CHO_high_ diets provides evidence that in men and women with abdominal obesity, almond supplementation in amounts representing 20% E does not attenuate features of atherogenic dyslipidemia induced by higher carbohydrate feeding. Thus, consumption of almonds should not be considered a means of improving this dyslipidemia in the setting of higher CHO intake.

## Additional file

Additional file 1: **Table S1.** Habitual dietary intake of study participants. **Table S2.** Statistical analyses of body weight, blood pressure, lipoproteins, and insulin resistance during a crossover study in 24 men and women. **Table S3**. Mean weight, blood pressure, lipoproteins and insulin resistance at the end of each diet. **Table S4.** Statistical analyses of interleukin, resistin, tumor necrosis factor alpha (TNF-α), plasminogen activator inhibitor-1 (PAI-1), leptin, monocyte chemotactic protein (MCP), serum amyloid A (SAA), lipocalin, and B lymphocyte activating factor (BAFF) during a crossover study in 24 men and women. **Table S5**. Mean differences in interleukin, resistin, tumor necrosis factor alpha (TNF-α), plasminogen activator inhibitor-1 (PAI-1), leptin, monocyte chemotactic protein (MCP), serum amyloid A (SAA), lipocalin, and B lymphocyte activating factor (BAFF) between diets. **Table S6**. Mean interleukin, resistin, tumor necrosis factor alpha (TNF-α), plasminogen activator inhibitor-1 (PAI-1), leptin, monocyte chemotactic protein (MCP), serum amyloid A (SAA), lipocalin, and B lymphocyte activating factor (BAFF) at the end of each diet. (PDF 377 kb)

## Abbreviations

CHO: Carbohydrate
CHO_high + almonds_: Higher-CHO diet with isocaloric substitution of 20%E from almonds
CHO_high_: Higher-carbohydrate reference diet
CHO_low_: Lower-CHO reference diet
HDL: High density lipoproteins
IDL: Intermediate density lipoproteins
LDL: Low density lipoproteins
MUFA: Monounsaturated fatty acids
PUFA: Polyunsaturated fatty acids
VLDL: Very low density lipoproteins
