# Supplementary material for: A randomized, controlled trial on the effects of almonds on lipoprotein response to a higher carbohydrate, lower fat diet in men and women with abdominal adiposity
Source: Lipids Health Dis. 2019 Apr 3;18:83. doi: 10.1186/s12944-019-1025-4 (PMC6446393; doi:10.1186/s12944-019-1025-4)
Supplement: Supplementary file 1 — Table S1. Habitual dietary intake of study participants. Table S2. Statistical analyses of body weight, blood pressure, lipoproteins, and insulin resistance during a crossover study in 24 men and women. Table S3. Mean weight, blood pressure, lipoproteins and insulin resistance at the end of each diet. Table S4. Statistical analyses of interleukin, resistin, tumor necrosis factor alpha (TNF-α), plasminogen activator inhibitor-1 (PAI-1), leptin, monocyte chemotactic protein (MCP), serum amyloid A (SAA), lipocalin, and B lymphocyte activating factor (BAFF) during a crossover study in 24 men and women. Table S5. Mean differences in interleukin, resistin, tumor necrosis factor alpha (TNF-α), plasminogen activator inhibitor-1 (PAI-1), leptin, monocyte chemotactic protein (MCP), serum amyloid A (SAA), lipocalin, and B lymphocyte activating factor (BAFF) between diets. Table S6. Mean interleukin, resistin, tumor necrosis factor alpha (TNF-α), plasminogen activator inhibitor-1 (PAI-1), leptin, monocyte chemotactic protein (MCP), serum amyloid A (SAA), lipocalin, and B lymphocyte activating factor (BAFF) at the end of each diet. (PDF 377 kb) [file 12944_2019_1025_MOESM1_ESM.pdf]

A randomized, controlled trial on the effects of almonds on lipoprotein response to a higher carbohydrate, lower fat diet in men and women with abdominal adiposity

Paul T. Williams, Nathalie Bergeron, Sally Chiu, Ronald M. Krauss

S1 Table. Habitual dietary intake of study participants

% Energy (kcal)	Females		Males	
	Mean	SD	Mean	SD
Calories	1797	847	2137	748
Carbohydrate	48	6	49	7
Protein	16	2	15	2
Total fat	37	6	37	6
SFA	11	2	12	2
MUFA	15	3	15	2
PUFA	9	2	7	2
Cholesterol (mg/d)	222	113	313	171
Sodium (mg/d)	3144	1718	3440	998
Fiber (g/d)	21	10	17	6

Habitual dietary intakes estimated by Block Food Frequency Questionnaire

S2 Table. Statistical analyses of body weight, blood pressure, lipoproteins, and insulin resistance during a crossover study in 24 men and women.

	Significance of cross-over study effects (P)			
	Treatment	Period	Carry-forward	Sequence
Body mass (kg)	0.26	0.40	0.95	0.56
Body mass index (kg/m^2)	0.27	0.55	0.89	0.4
Waist circumference(cm)	0.92	0.25	0.26	0.13
Systolic blood pressure (mmHg)	0.21	0.61	0.30	0.17
Diastolic blood pressure (mmHg)	0.01	0.02	0.02	0.49
Triglycerides (mg/dL)	0.77	0.26	0.10	0.09
Total cholesterol (mg/dl)	0.16	0.09	0.47	0.82
LDL-cholesterol (mg/dl)	0.22	0.10	0.02	0.54
HDL-cholesterol (mg/dl)	0.31	0.77	0.49	0.63
nonHDL-cholesterol (mg/dl)	0.23	0.12	0.26	0.42
Total-/HDL-cholesterol	0.64	0.48	0.40	0.33
Glucose (mg/dL)	0.36	0.70	0.55	0.96
Insulin (pmol/L)	0.35	0.29	0.15	0.67
Homa-IR	0.32	0.31	0.11	0.63
Apolipoprotein AI (mg/dL)	0.07	0.10	0.06	0.38
Apolipoprotein B (mg/dL)	0.18	0.47	0.15	0.66
HDL3&2a (nmol/L)	0.60	0.98	0.19	0.47
HDL2b (nmol/L)	0.46	0.43	0.23	0.41
IDL (nmol/L)	0.63	0.84	0.67	0.28
LDL IVc (nmol/L)	0.59	0.98	0.90	0.53
LDL IVb (nmol/L)	0.59	0.6	0.78	0.38
LDL IVa (nmol/L)	0.53	0.77	0.98	0.67
LDL IIIb (nmol/L)	0.33	0.62	0.86	0.60
LDL IIIa (nmol/L)	0.06	0.69	0.99	0.31
LDL IIb (nmol/L)	0.02	0.10	0.10	0.34
LDL IIa (nmol/L)	0.34	0.03	0.02	0.75
LDL I (nmol/L)	0.81	0.05	0.02	0.45
IDL2 (nmol/L)	0.44	0.07	0.02	0.22
IDL1 (nmol/L)	0.19	0.12	0.65	0.89
Small VLDL (nmol/L)	0.65	0.35	0.84	0.65
Intermediate VLDL (nmol/L)	0.19	0.85	0.40	0.23
Large VLDL (nmol/L)	0.20	0.92	0.65	0.41
LDL peak diameter (nmol/L)	0.12	0.19	0.10	0.28

P-values calculated using ANOVA for a cross-over design

S3 Table. Mean weight, blood pressure, lipoproteins and insulin resistance at the end of each diet.

	$CHO_{High+almonds}$		CHO_{High}		CHO_{Low}	
	Mean	SE	Mean	SE	Mean	SE
Body mass (kg)	87.8	0.2	88.0	0.2	87.4	0.2
Body mass index (kg/m^2)	30.8	0.1	30.9	0.1	30.7	0.1
Waist circumference (cm)	101.3	0.4	100.9	0.4	100.6	0.4
Systolic BP (mmHg)	118	1	119	1	118	1
Diastolic BP (mmHg)	69	1	70	1	68	1
Triglycerides (mmol/L)	1.35	0.14	1.32	0.15	1.33	0.14
Total cholesterol (mmol/L)	4.52	0.07	4.58	0.07	4.42	0.07
LDL-cholesterol (mmol/L)	2.64	0.07	2.62	0.08	2.6	0.08
HDL-cholesterol (mmol/L	1.26	0.04	1.34	0.04	1.27	0.04
nonHDL-cholesterol (mmol/L)	3.26	0.08	3.24	0.08	3.15	0.08
Total-/HDL-cholesterol	3.8	0.1	3.8	0.1	3.7	0.1
Glucose (mmol/L)	5.9	0.1	5.8	0.1	5.9	0.1
Insulin (pmol/L)	22.2	1.2	20.6	1.2	21.7	1.2
HOMA-IR	5.9	0.3	5.4	0.3	5.7	0.3
Apolipoprotein AI (g/L)	1.30	0.03	1.35	0.03	1.31	0.03
Apolipoprotein B (g/L)	0.79	0.02	0.79	0.02	0.76	0.02
HDL3&2a (nmol/L)	20910	284	20810	289	20341	279
HDL2b (nmol/L)	7880	226	8147	231	7898	223
LDL IVc (nmol/L)	84.8	1.8	85.4	1.8	82.9	1.8
LDL IVb (nmol/L)	73.4	3.6	69.5	3.6	65.7	3.5
LDL IVa (nmol/L)	98.2	7.3	89.5	7.5	84.2	7.2
LDL IIIb (nmol/L)	84.9	4.0	77.4	4.1	74.6	4.0
LDL IIIa (nmol/L)	203.0	7.4	192.0	7.6	174.4	7.3
LDL IIb (nmol/L)	234.2	8.0	238.0	8.1	213.2	7.9
LDL IIa (nmol/L)	209.8	7.7	216.8	7.9	209.6	7.6
LDL I (nmol/L)	286.1	11.3	287.8	11.5	307.5	11.1
IDL2 (nmol/L)	219.7	7.3	213.8	7.4	225.4	7.1
IDL1 (nmol/L)	165.4	5.0	161.1	5.1	154.1	4.9
Small VLDL (nmol/L)	65.4	2.3	64.0	2.3	62.1	2.2
Intermediate VLDL (nmol/L)	60.0	2.7	60.7	2.8	53.7	2.7
Large VLDL (nmol/L)	19.7	1.3	20.6	1.3	16.9	1.3
LDL peak diameter (Å)	220.1	0.4	220.7	0.5	221.8	0.4

S4 Table. Statistical analyses of interleukin, resistin, tumor necrosis factor alpha (TNF-α), plasminogen activator inhibitor-1 (PAI-1), leptin, monocyte chemotactic protein (MCP), serum amyloid A (SAA), lipocalin, and B lymphocyte activating factor (BAFF) during a crossover study in 24 men and women.

	Significance of cross-over study effects (P)			
	Treatment	Period	Carry- forward	Sequence
Interleukin-1 (pg/ml)	0.29	0.26	0.75	0.99
Interleukin-6 (pg/ml)	0.21	0.07	0.54	0.99
Interleukin-8 (pg/ml)	0.34	0.68	0.70	0.78
Interleukin-10 (pg/ml)	0.73	0.01	0.02	0.77
Resistin (pg/ml)	0.91	0.71	0.99	0.66
TNF-alpha (pg/ml)	0.41	0.71	0.88	0.99
PAI-1 (pg/ml)	0.06	0.12	0.08	0.14
Leptin (pg/ml)	0.30	0.10	0.74	0.99
MCP (pg/ml)	0.69	0.77	0.91	0.96
SAA (pg/ml)	0.74	0.01	0.04	0.88
Lipocalin (pg/ml)	0.19	0.06	0.21	0.22
BAFF (pg/ml)	0.88	0.22	0.81	0.88

P-values calculated using ANOVA for a cross-over design

S5 Table. Mean differences in interleukin, resistin, tumor necrosis factor alpha (TNF-α), plasminogen activator inhibitor-1 (PAI-1), leptin, monocyte chemotactic protein (MCP), serum amyloid A (SAA), lipocalin, and B lymphocyte activating factor (BAFF) between diets.

	Mean and SE for differences between diets					
	$CHO_{High+almonds}$- CHO_{High}		$CHO_{High+almonds}$- CHO_{Low}		CHO_{High}- CHO_{Low}	
	Mean	SE	Mean	SE	Mean	SE
Interleukin-1 (pg/ml)	3.7	2.3	4.6*	2.3	0.9	2.3
Interleukin-6 (pg/ml)	0.8	2.1	3.1	2.0	2.3	2.0
Interleukin-8 (pg/ml)	12.0	8.3	5.1	8.2	-6.9	8.2
Interleukin-10 (pg/ml)	0.05	0.17	0.04	0.17	-0.01	0.17
Resistin (pg/ml)	357	988	-14	972	-371	972
TNF-alpha (pg/ml)	4.1	4.1	5.0	4.1	0.9	4.1
PAI-1 (pg/ml)	-387	469	370	462	757	462
Leptin (pg/ml)	447	489	766	481	319	481
MCP (pg/ml)	9.4	13.1	8.2	12.9	-1.2	12.9
SAA (pg/ml)	-774	10609	6099	10440	6873	10440
Lipocalin (pg/ml)	2668	2441	-1000	2402	-4000	2402
BAFF (pg/ml)	2.7	5.8	-0.8	5.7	-3.5	5.7

Significance levels coded: * $P \leq 0.05$

S6 Table. Mean interleukin, resistin, tumor necrosis factor alpha (TNF-), plasminogen activator inhibitor-1 (PAI-1), leptin, monocyte chemotactic protein (MCP), serum amyloid A (SAA), lipocalin, and B lymphocyte activating factor (BAFF) at the end of each diet.

	$CHO_{High+almonds}$		CHO_{High}		CHO_{Low}	
	Mean	SE	Mean	SE	Mean	SE
Interleukin-1 (pg/ml)	59.1	1.6	55.4	1.6	54.5	1.6
Interleukin-6 (pg/ml)	58.2	1.5	57.4	1.4	55.1	1.4
Interleukin-8 (pg/ml)	115.3	5.8	103.3	5.7	110.2	5.7
Interleukin-10 (pg/ml)	4.25	0.12	4.20	0.12	4.21	0.11
Resistin (pg/ml)	33182	696	32825	681	33196	674
TNF-alpha (pg/ml)	86.9	2.9	82.8	2.8	81.9	2.8
PAI-1 (pg/ml)	4196	330	4582	323	3825	320
Leptin (pg/ml)	8037	344	7590	337	7271	333
MCP (pg/ml)	243.7	9.2	234.3	9.0	235.6	9.0
SAA (pg/ml)	146959	7473	147734	7315	140861	7239
Lipocalin (pg/ml)	95857	1719	93189	1683	97279	1665
BAFF (pg/ml)	258.3	4.1	255.5	4.0	259.1	3.9
